# Advances in Lung Cancer Therapy

**DOI:** 10.3390/cancers15102671

**Published:** 2023-05-09

**Authors:** Domenico Galetta

**Affiliations:** 1 Division of Thoracic Surgery, European Institute of Oncology IRCCS, 20141 Milan, Italy; domenico.galetta@ieo.it; Tel.: +39-0257489801; 2 Department of Oncology and Hematology-Oncology-DIPO, University of Milan, 20122 Milan, Italy

Lung cancer, including both small cell lung cancer (SCLC) and non-small cell lung cancer (NSCLC), remains one of the most aggressive types of cancer, and the prognosis for individuals diagnosed with this neoplasm has, for the most part, been insufficient. However, the last decade has seen important advances both in diagnosis and treatment (surgery, chemotherapy, radiation therapy, and personalized therapy—immune and targeted therapies), which have led to important improvements in lung cancer survival rates.

This series of 17 articles (12 research papers, 4 reviews, and 1 review and meta-analysis) is presented by international leaders in the field of lung cancer and deals with different aspects of the diagnosis and treatment of this tumor. 

Rodak and colleagues [1] summarized the recent advances in NSCLC focusing on epidemiology, the latest histopathological classification, lung cancer heterogeneity, targeted therapy, and immunotherapy. They also reviewed the treatment perspectives in targeted therapy of the best-known genotypes of NSCLC, and also reported a comprehensive summary of the current immunotherapies and the predictive biomarkers approved by the FDA and ongoing clinical trials.

Liao et al. [2] investigated the effects of Fasudil, a selective inhibitor of Rho kinase approved by the Japanese and Chinese governments for the clinical treatment of cerebral vasospasm, on the proliferation and apoptosis of TKI-sensitive mutations and TKI-resistant mutant NSCLC cells. They also evaluated the anti-tumor effects of a novel treatment strategy involving the co-administration of Fasudil and an EGFR-TKI (gefitinib) in gefitinib-resistant EGFR-mutated NSCLC using in vivo and in vitro models, showing that Fasudil could effectively inhibit EGFR-mutated cell growth and enhance the sensitivity of gefitinib-resistant NSCLC cells to gefitinib by suppressing intracellular lipid accumulation.

Jiang et al. [3], using The Cancer Genome Atlas, the GEO database, the Cancer Cell Line Encyclopedia, and the Genomics of Drug Sensitivity in Cancer databases, attempted to identify candidate genes associated with erlotinib resistance and cancer stemness in lung adenocarcinoma. The authors constructed an erlotinib resistance model using candidate genes and analyzed the correlation between erlotinib resistance genes and stemness, and between erlotinib resistance genes and the tumor microenvironment, through various bioinformatics methods. Moreover, by means of in vitro experiments, the authors demonstrated that NCAPG2 maintained cancer stemness and promoted erlotinib resistance in lung adenocarcinoma cells, providing new insights into diagnosing and treating lung adenocarcinoma with erlotinib resistance. 

Misri and colleagues [4] report the inhibitory properties of cannabidiol, a non-psychoactive phytochemical derived from the cannabis plant, on the growth and metastasis of cisplatin-resistant NSCLC in vitro and in vivo compared to cisplatin treatment. The authors showed that cannabidiol induces apoptotic signals, together with increased reactive oxygen species in cisplatin-resistant cells in a transient receptor potential vanilloid-2 (TRPV2)-dependent manner. High expression of TRPV2 was found in NSCLC cell lines and in lung adenocarcinoma patients, where it was associated with poor overall survival. This study provided novel insights into the anti-tumor effects mediated by cannabidiol CBD in cisplatin-resistant NSCLC.

Refeno et al. [5] investigated systemic treatments beyond the second line in an EGFR-mutated population and analyzed the survival of EGFR-mutated patients treated beyond the second line of treatment. The authors reported that for EGFR-mutated patients who received TKI in the first line and chemotherapy in the second line, TKI appeared to be a better alternative in the third line compared to chemotherapy. Osimertinib may be used in third-line treatment if not used before.

Gagiannis and collaborators [6] investigated whether polysialic acid (polySia) expression, usually localized on the neuronal cell adhesion molecule (NCAM), has an impact on the disease progression, treatment response, and prognosis of lung neuroendocrine tumors. The authors analyzed tissue samples from 28 patients via immunohistochemistry for the presence of polySia-NCAM and concluded that NCAM-polySia is not very useful as a prognostic factor for poor disease outcome. However, it was still interesting as a therapeutic target for individual tumor therapy, as a majority of the patients (78.6%) showed a strong staining signal for NCAM-polySia.

Spini et al. [7] described first-line pharmacotherapy and overall survival in non-resectable non-small cell lung cancer (nrNSCLC) patients by gender. The authors highlighted a different use of target therapies between sexes: in women with non-squamous NSCLC, anti-EGFR medications were the most frequently used target drugs according to the known biomolecular profile of females. In patients receiving a first-line treatment, survival seemed to slightly improve over the study period for both histologies, although a poor reduction in the mortality risk was still observed. Male and female patients also presented a different survival profile when diagnosed with squamous or non-squamous NSCLC.

Ahn and colleagues [8] evaluated the efficacy and safety of 80 mg osimertinib orally, once daily, in a preplanned, exploratory analysis of the central nervous system (CNS) activity of osimertinib in the Korean subgroup of the ASTRIS trial [9] and confirmed the consistent CNS bioavailability of osimertinib with fully matured OS and PFS for T790M-positive NSCLC, with disease progression with first-line EGFR-TKI.

Su and coworkers [10] evaluated the survival impact of diabetes severity on lung cancer. They performed head-to-head propensity score matching to estimate the survival impact of various Adapted Diabetes Complications Severity Index (aDCSI) scores among patients with both diabetes and lung squamous cell carcinoma (SqCLC). The results of their study revealed that severe diabetes (aDCSI ≥ 2) was an independent prognostic factor for OS among patients with both diabetes and lung SqCLC who receive standard treatments. Preventing diabetes progression is necessary for patients with diabetes because it not only supports diabetes control but also improves survival for patients with lung SqCLC.

Two studies focused on immune checkpoint inhibitors [11,12]. In a retrospective study, Xie et al. [11] evaluated the efficacy of programmed death-1 (PD-1)/programmed death ligand-1 (PD-L1) inhibitors in NSCLC patients with liver metastases. They showed that PD-1/PD-L1 inhibitors are effective in NSCLC patients with liver metastases but were inferiorly effective in patients without liver metastases. In addition, PD-L1 expression and CD8+ T-cell infiltration may be potential biomarkers for PD-1/PD-L1 inhibitor therapy in NSCLC patients with liver metastases.

Passaro and colleagues [12] performed a multicenter retrospective study in five Italian centers on patients diagnosed with metastatic NSCLC expressing high PD-L1 levels (≥50%) who received pembrolizumab monotherapy as a first-line approach. The objective of the study was to identify clinical factors and develop a prognostic score model predicting the probability of developing early progression (EP), defined as progression of the disease within three months from pembrolizumab initiation. The authors identified six clinical factors independently associated with EP and developed a prognostic score model for EP risk to potentially improve clinical practice and patient selection for 1L pembrolizumab in NSCLC with high PD-L1, in the real-world clinical setting.

Three studies focused on the surgical aspects in the treatment of advanced lung cancer [13,14,15].

Galetta and co-authors [13] analyzed and reported the surgical and long-term outcomes of patients with initial unresectable, locally advanced, or oligometastatic NSCLC who were treated with TKIs or ICIs achieving a clinical downstaging so as to re-enter resectability. The authors showed that lung resection for suspected residual disease after immunotherapy or TKIs was feasible, with encouraging pathological downstaging. Moreover, surgical operation was sometimes technically challenging due to the presence of fibrosis, but significant morbidity appeared to be rare. Galetta reported that the outcomes are encouraging, with reasonable survival during the short-interval follow-up.

In another study, Galetta and Spaggiari [14] focused on the role of induction therapy (IT) in the outcomes of patients who have undergone completion pneumonectomy (CP), reviewing their single-center experience in patients receiving CP for recurrent/second NSCLC after IT, and they analyzed perioperative results and long-term outcomes. Their results pointed out that CP had low mortality, acceptable morbidity, and good long-term survival, which justifies this surgical procedure. Postoperative complications were not influenced by IT. Long-term survival was adversely influenced by the absence of IT, the presence of extended resection, the presence of squamous cell carcinoma, and cancers at advanced stages.

Menna et al. [15] performed a review on parenchymal sparing surgery for lung cancer, focusing on pulmonary artery (PA) reconstruction. In this review, the authors addressed some controversial aspects concerning the intraoperative and perioperative management of a sleeve resection with pulmonary artery reconstruction that may influence the outcome. Menna and colleagues underlined that the results from the main literature data confirmed the reliability of lobectomy associated with PA reconstruction in terms of perioperative morbidity and long-term survival, and also confirmed that sleeve lobectomy and PA reconstruction can be performed safely and effectively even after induction therapy.

Saman and colleagues [16] reviewed and assessed the effectiveness of different techniques—currently in use and that are upcoming—in the early detection of lung cancer. They presented and evaluated the principles of developing such techniques and how to overcome challenges frequently facing researchers in the field of early lung cancer detection.

Cortes-Dericks and Galetta [17] focused on the current knowledge of the existence of cancer stem cells (CSCs), CSC-associated mechanisms of chemoresistance, the ability of CSCs to evade immune surveillance, and potential CSC inhibitors in lung cancer, to provide a wider insight to drive a more efficient elimination of this pro-oncogenic and treatment-resistant cell fraction. 

Galata and co-authors [18] assessed the evidence of perioperative X-rays following thoracic surgery and estimated the clinical value with regard to changes in patient care. The authors pointed out that performing serial X-rays in the perioperative window of general thoracic surgery had several disadvantages. It was associated with increased workload, increased hospital costs, and, most importantly, exposure of the individual patient, further patients, and medical staff to radiation. In their analysis, there was no strong evidence to support performing any X-ray apart from the X-ray after having removed the pleural drain, showing that the probability of a perioperative X-ray having a relevant consequence is rather low. Thus, in their point of view, it is therefore reasonable to question each indication for an X-ray on an asymptomatic patient and think about the potential consequences that the X-ray would have.

In conclusion, in this Special Issue, an updated overview of the most important aspects of the diagnosis and treatment of lung cancer has been provided by 17 high-quality papers. I am confident that they may offer an updated tool to better understand and treat lung cancer.
